# A two-stage workflow for vitiligo diagnosis: clinical characteristic classification and large language model (LLM)–based report generation

**DOI:** 10.3389/fimmu.2026.1853327

**Published:** 2026-06-01

**Authors:** Kaiqiao He, Tianle Xu, Yining Feng, Yafei Lu, Xinju Wang, Linhan Jiang, Sen Guo, Yuanmin He, Wei Dai, Wei Zhang, Jianglin Zhang, Hongbing Lu, Dong Huang, Shuli Li

**Affiliations:** 1 Department of Dermatology, Xijing Hospital, Fourth Military Medical University, Xi’an, Shaanxi, China; 2 School of Biomedical Engineering, Fourth Military Medical University, Xi’an, Shaanxi, China; 3 Department of Dermatology, The Affiliated Hospital of Southwest Medical University, Luzhou, Sichuan, China; 4 Department of Dermatology, Nanfang Hospital, Southern Medical University, Guangzhou, Guangdong, China; 5 Hospital for Skin Diseases, Institute of Dermatology, Chinese Academy of Medical Sciences & Peking Union Medical College, Nanjing, Jiangsu, China; 6 Department of Dermatology, Shenzhen People’s Hospital (The Second Clinical Medical College, Jinan University; The First Affiliated Hospital, Southern University of Science and Technology), Shenzhen, Guangdong, China

**Keywords:** artificial intelligence, clinical report generation, differential diagnosis, large language model, vitiligo

## Abstract

**Background:**

Vitiligo is a common depigmenting disorder frequently misdiagnosed due to its visual similarity to other hypopigmentary conditions. While artificial intelligence (AI) has shown promise in dermatological image analysis, most models lack interpretability and fail to provide actionable clinical recommendations.

**Objective:**

To develop and validate an AI-assisted diagnostic system that integrates a large language model (LLM) for differentiating vitiligo from ten other hypopigmentary disorders, while providing interpretable characteristics and structured clinical reports.

**Methods:**

We retrospectively collected clinical images from a multicenter cohort, including patients diagnosed with vitiligo or one of ten other hypopigmentary disorders across five hospitals in China. A multi-task Vision Transformer model was trained to classify eight key clinical characteristics and output diagnostic probabilities. The model’s structured predictions were then fed into the DeepSeek LLM to generate comprehensive clinical reports. Diagnostic performance was evaluated on an independent set of 175 images (87 vitiligo and 88 non-vitiligo) and compared with 43 dermatologists.

**Results:**

The study included 13,322 clinical images from 2,974 patients. For distinguishing vitiligo, the model achieved an AUC of 0.9906 (95% CI: 0.9844–0.9968), with sensitivity of 98.29% and specificity of 93.73%. Through multi-task learning, the model demonstrated accurate classification of eight key clinical characteristics, notably achieving 88.12% accuracy in identifying typical location and 86.78% in recognizing edge morphology. In a comparative test using 175 independent images, the AI model (AUC = 0.98) significantly outperformed a panel of dermatologists, particularly in diagnostic sensitivity. Moreover, the system successfully generated clinical reports via the DeepSeek LLM, providing structured diagnostic suggestions, differential diagnoses, and treatment plans.

**Conclusion:**

This multicenter study presents a clinically interpretable AI system for accurately discriminating vitiligo from similar disorders. By generating LLM-based reports, it enhances diagnostic transparency and supports clinical decision-making, offering potential assistance especially in resource-limited settings and advancing intelligent tools in dermatology.

## 1 Introduction

Vitiligo, the most common depigmentation skin disorder, is characterized by a chronic, recurrent, and refractory nature, which not only affects patients’ appearance but also imposes significant psychological and social burdens, markedly diminishing their quality of life (1, 2). Its diagnosis is frequently complicated by other conditions that manifest as skin depigmentation or hypopigmentation, such as nevus depigmentosus, nevus anemicus and pityriasis alba (3, 4). The diagnosis of these diseases primarily relies on the clinical experience of dermatologists, supplemented by auxiliary tools like Wood’s lamp, dermoscopy, and histopathological biopsy (5). It is reported that approximately 44.9% of vitiligo patients worldwide have been misdiagnosed, closely linked to disparities in medical resources and geographical location (6). Therefore, the accuracy of differential diagnosis is critical as it directly influences treatment strategies and patient prognosis.

Artificial intelligence (AI), particularly deep learning, has achieved significant breakthroughs in medical image analysis. In dermatology, AI can extract subtle characteristics from large image datasets that are imperceptible to the human eye, enabling precise classification and diagnosis of skin diseases (7). These models have matched or even surpassed human experts in the early detection of skin cancer and inflammatory skin diseases (8–11). While AI has shown promising potential in diagnosing vitiligo and other hypopigmentary disorders, current research faces several challenges (12–14). First, the scope of these studies is often narrow, lacking systematic comparisons between vitiligo and other hypopigmentary disorders. Second, small-sample designs limit model generalizability and robustness. Third, the “black-box” nature of many AI models, which output only disease labels without providing diagnostic rationale or management guidance, restricts their role in complex clinical decision-making and hinders the full trust of clinicians.

To address these research gaps and advance AI from simple classification to intelligent clinical decision support, we propose an intelligent diagnostic system that integrates deep learning with a large language model (LLM). The system utilizes a high-precision deep learning model to automatically extract lesion characteristics (such as location and border) and output the diagnostic probability for vitiligo and other hypopigmentary disorders. To our knowledge, this is the first study in dermatology to integrate an LLM for generating structured reports that include diagnostic opinions, differential analyses, treatment suggestions, and follow-up plans. This approach overcomes the “black-box” limitation of traditional AI and explores the feasibility and reliability of LLMs in generating clinical reports.

## 2 Materials and methods

### 2.1 Study design

**Figure 1:**
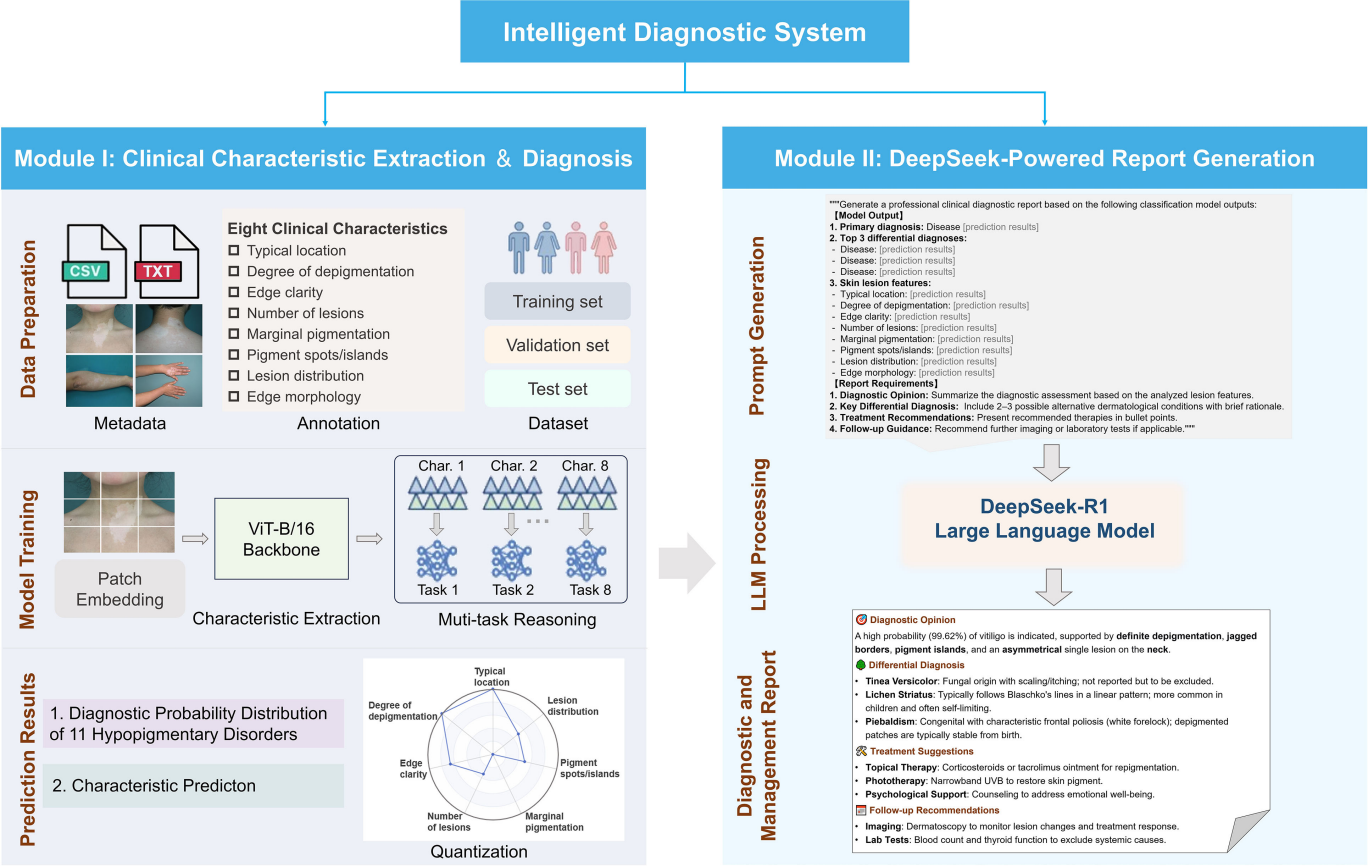
Schematic overview of the two-stage intelligent diagnostic system. The workflow comprises two integrated modules. Module I (Clinical Characteristic Extraction & Diagnosis): Clinical images of hypopigmentary disorders are annotated for eight key diagnostic characteristics and used to train a multi-task Vision Transformer (ViT-B/16) model. This model extracts visual characteristics, performs simultaneous characteristic classification, and outputs both a probability score for hypopigmentary disorders diagnosis and predictions for individual clinical characteristics. The results are visualized via a radar chart for interpretability. Module II (DeepSeek-Powered Report Generation): The structured output from Module I (diagnosis probability, characteristic predictions) serves as input to the DeepSeek-R1 large language model (LLM). Guided by a predefined prompt template, the LLM synthesizes this information to automatically generate a comprehensive clinical report, which includes diagnostic conclusions, differential diagnoses, treatment suggestions, and follow-up recommendations.

This study employed a multi-stage hybrid design, beginning with the retrospective collection and annotation of clinical images and metadata. Subsequently, a deep learning model based on multiple Vision Transformers and multi-task reasoning was trained and optimized on this dataset to generate differential diagnoses. Finally, the model’s structured output was fed into the DeepSeek LLMs to automatically generate comprehensive clinical reports, including diagnostic analyses, treatment plans, and follow-up recommendations (outlined in **Figure 1**).

### 2.2 Study population

This study retrospectively enrolled patients from five hospitals across different geographic regions of China between January 2019 and December 2024, including all eligible cases. Participants were diagnosed with either vitiligo or one of ten other clinical similar hypopigmentary disorders: lichen sclerosus, lichen striatus, nevus depigmentosus, nevus anemicus, idiopathic guttate hypomelanosis (IGH), pityriasis alba, piebaldism, tinea versicolor, hypopigmented mycosis fungoides (MF), and two forms of hereditary dyschromatosis, including dyschromatosis universalis hereditaria (DUH) and dyschromatosis symmetrica hereditaria (DSH). The initial clinical diagnosis was established by at least two dermatologists based on comprehensive clinical assessment and Wood’s lamp examination (15). All cases were subsequently confirmed by histopathological evaluation to ensure diagnostic certainty for model training. Patients were excluded if they had an ambiguous diagnosis, poor-quality clinical images, a history of treatments that could interfere with the evaluation, or co-existing confounding dermatological conditions.

### 2.3 Ethical approval and data source

Clinical images and associated metadata from patients diagnosed with hypopigmentary disorders were retrospectively collected. Written informed consent was obtained from all participants (or their legal guardians) for the use of their clinical images and data. This study was conducted in accordance with the Declaration of Helsinki and approved by the Ethics Committee of Xijing Hospital, Fourth Military Medical University (KY20222324-C-1). All procedures involving human participants complied with relevant ethical standards, and all data were anonymized to protect patient privacy.

#### 2.3.1 Image acquisition and standardization

Clinical images were acquired from five participating hospitals using high-resolution digital cameras. While lighting conditions and backgrounds varied across sites, all images were converted to a standardized format and resolution, including format conversion, cropping and basic color correction, to reduce technical variability. Multiple images were captured per patient to document different lesions, with corresponding clinical details stored in.txt and.csv files.

#### 2.3.2 Characteristic annotation

To construct a clinically interpretable dataset, each image was annotated by experienced dermatologists based on eight key clinical characteristics: typical location, degree of depigmentation, edge clarity, number of lesions, marginal pigmentation, pigment spots/islands, lesion distribution, and edge morphology. The clinical significance is provided in the **Supplementary Material**, and their detailed classifications are provided in **Table 1**.

**Table 1:** Summary of the eight annotated clinical characteristics and their classification criteria.

Clinical characteristics	Classification type	Number of categories	Definition and Description
Typical location	Multi-class classification	16	Face, Neck, Scalp, Dorsum of Hands, Axillae, Elbows, Wrists, Knees, Lower Back, Groin, Inframammary Area, Lips, Genital Mucosa, Fingers, Toes, Atypical/Other Sites.
Degree of depigmentation	Multi-class classification	3	Definite, Indeterminate, Absent
Edge clarity	Multi-class classification	4	Distinct, Indistinct, Partially distinct, Cannot be assessed
Number of lesions	Multi-class classification	4	Single, 2-3, ≥4, Cannot be assessed
Marginal pigmentation	Multi-class classification	3	Definite, Indeterminate, Absent
Pigment spots/islands	Multi-class classification	3	Definite, Indeterminate, Absent
Lesion distribution	Multi-class classification	3	Symmetrical, Asymmetrical, Cannot be assessed
Edge morphology	Multi-label classification	8	Smooth, Jagged, Irregular, Linear, Confetti-like, Inflammatory, Sclerotic, Cannot be Assessed

#### 2.3.3 Data integration and dataset partitioning

Following annotation, the image data and corresponding labels for each patient were compiled into a structured JSON record containing the patient ID, image paths, and expert annotations. To prevent data leakage, the dataset was partitioned at the patient level, ensuring all images from a given patient remained within the same subset. Using stratified random sampling based on diagnosis, the data were split into a training set (70%) and a validation set (30%). No images from the same patient appeared in both the training and validation sets. Further training details are provided in the **Supplementary Material**.

### 2.4 Model architecture design

**Figure 2:**
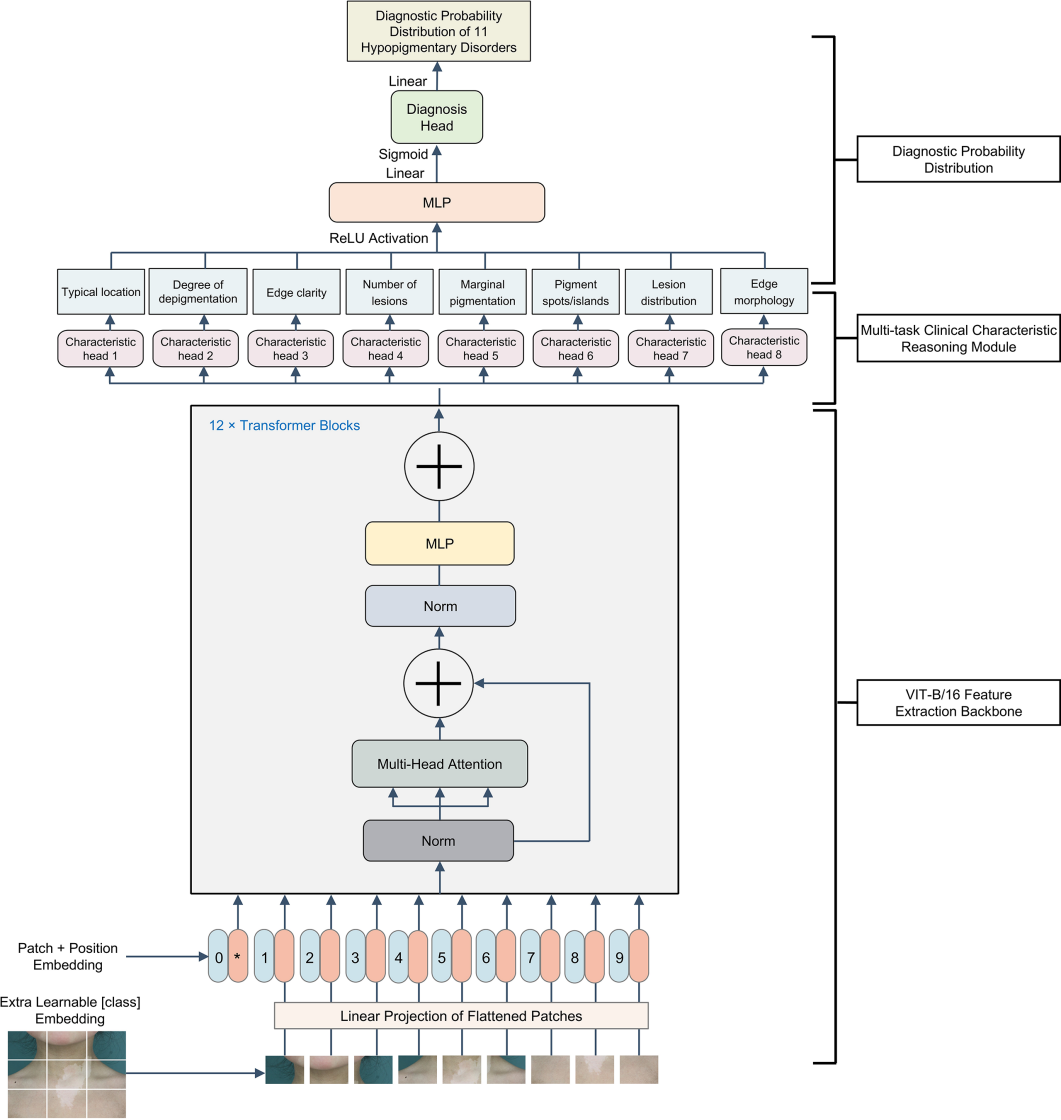
Architecture of the three-level cascade model for differential diagnosis of vitiligo and other hypopigmentary disorders. The model first utilizes a ViT-B/16 backbone to extract global image characteristics. These characteristics are then fed into a multi-task characteristic reasoning module to infer key clinical characteristics. Finally, based on the reasoned characteristics, the model outputs the diagnostic probabilities distribution of 11 hypopigmentary disorders.

The model employs a three-tier cascade structure (**Figure 2**). An input image is processed by a Vision Transformer (ViT-B/16) to extract visual features. These features enter a multi-task reasoning module that simultaneously classifies eight clinical characteristics. The module then synthesizes these predictions to output a diagnostic probability distribution across all 11 disorders. This explicit stepwise design enhances model interpretability by mirroring clinical reasoning. Further implementation details are provided in the **Supplementary Material**.

### 2.5 Clinical characteristic quantification

To enhance the interpretability of the model’s predictions, we developed a quantitative visualization method using a radar chart. Of the eight clinical characteristics annotated for vitiligo lesions, seven single-label characteristics were converted to numerical values, normalized via Min−Max scaling, and plotted in a radar chart (**Supplementary Figure 1**). Edge morphology, a multi-label characteristic, was excluded due to its non−scalar nature. This approach allows clinicians to intuitively assess the profile of characteristics for patients. Detailed quantification criteria for each characteristic are provided in **Supplementary Table 1**.

### 2.6 Report generation

The model’s outputs, including the diagnostic probability distribution across the 11 hypopigmentary disorders, the seven classified characteristics, and the multi-label edge morphology labels, are formatted into structured JSON format. This JSON data is then input into the DeepSeek R1 model via a predefined prompt (listed in **Supplementary Material**) that incorporates medical reasoning and reporting guidelines. The prompt directs the LLM to generate a comprehensive clinical report containing diagnostic opinions, differential diagnoses, treatment recommendations, and follow-up suggestions.

### 2.7 Evaluation of LLM-generated reports

To assess the clinical quality of LLM-generated structured reports independent of the image classifier, we conducted a validation study. Thirty cases (15 vitiligo, 15 non−vitiligo) were randomly selected from the test set. Three vitiligo specialists (none involved in model development or annotation) blindly evaluated the LLM reports on a 5−point Likert scale (1=very poor/unacceptable, 5=excellent/fully acceptable) across four dimensions: diagnostic accuracy, differential diagnosis plausibility, treatment appropriateness, and follow−up reasonableness. Mean score and standard deviation were calculated per dimension. Inter−rater reliability was assessed using the two−way random, absolute−agreement intraclass correlation coefficient (ICC), with values >0.75 indicating good agreement.

### 2.8 Human vs. AI diagnostic performance

An independent test set of 175 patient images (87 vitiligo, 88 non-vitiligo), not included in the 13,322 images used for training and validation, was used to compare the AI model with dermatologists. Through an anonymous online survey, dermatologists performed binary classification (vitiligo vs. non-vitiligo) based on images alone; their professional background (title, hospital level, specialty) was recorded for subgroup analysis. Accuracy, sensitivity, and specificity were calculated for both the AI model and the clinicians. Their discriminative abilities were assessed using Receiver Operating Characteristic (ROC) curve analysis and the Area Under the Curve (AUC). Subgroup differences were compared with t tests or ANOVA. All statistical analyses were performed using SPSS software (version 26.0), with a P-value of < 0.05 considered statistically significant.

## 3 Results

### 3.1 Patient and image cohort

**Figure 3:**
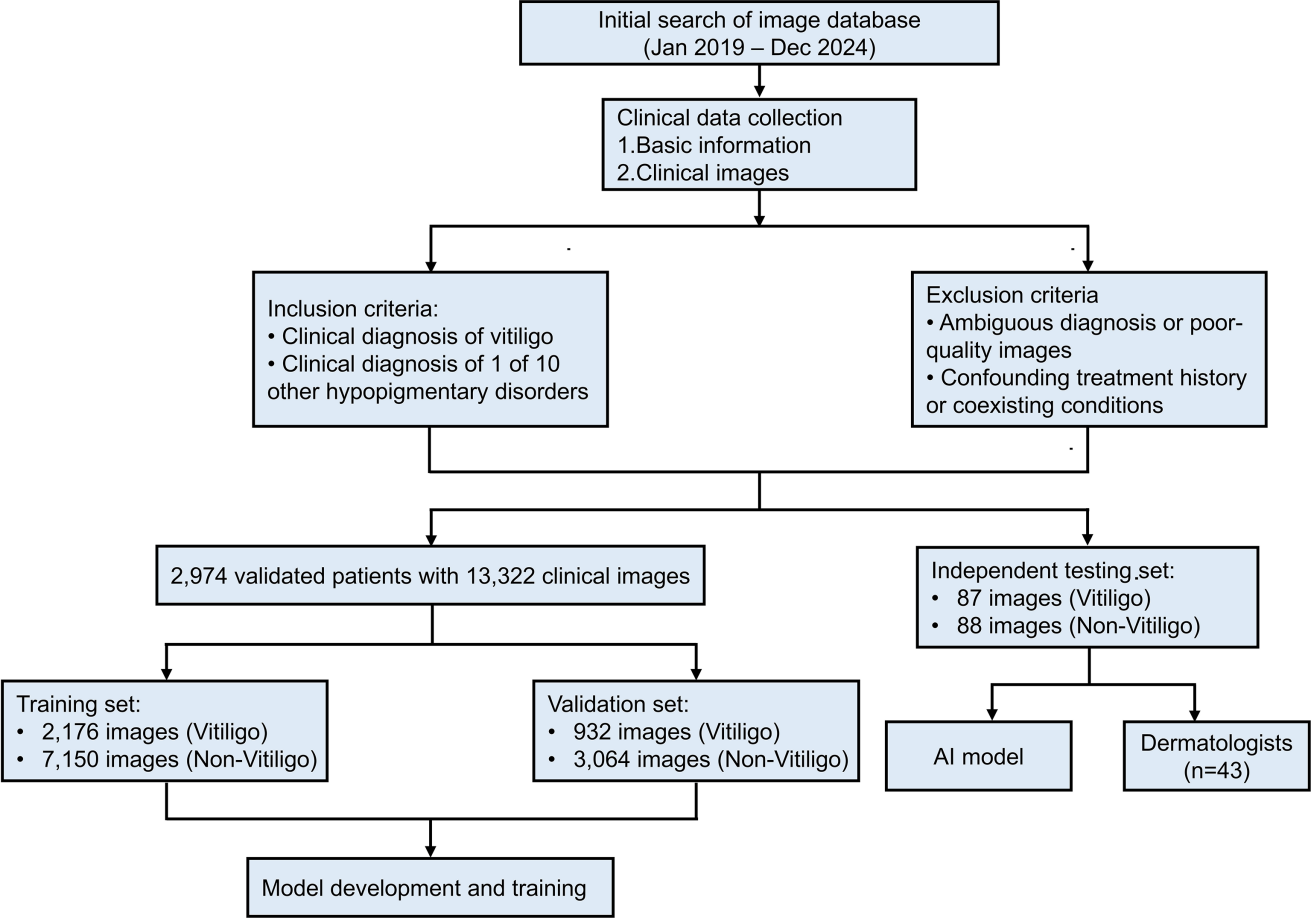
Flowchart showing the patient selection, image dataset partitioning, and development of an AI model for distinguishing vitiligo from other hypopigmentary disorders.

**Figure 4:**
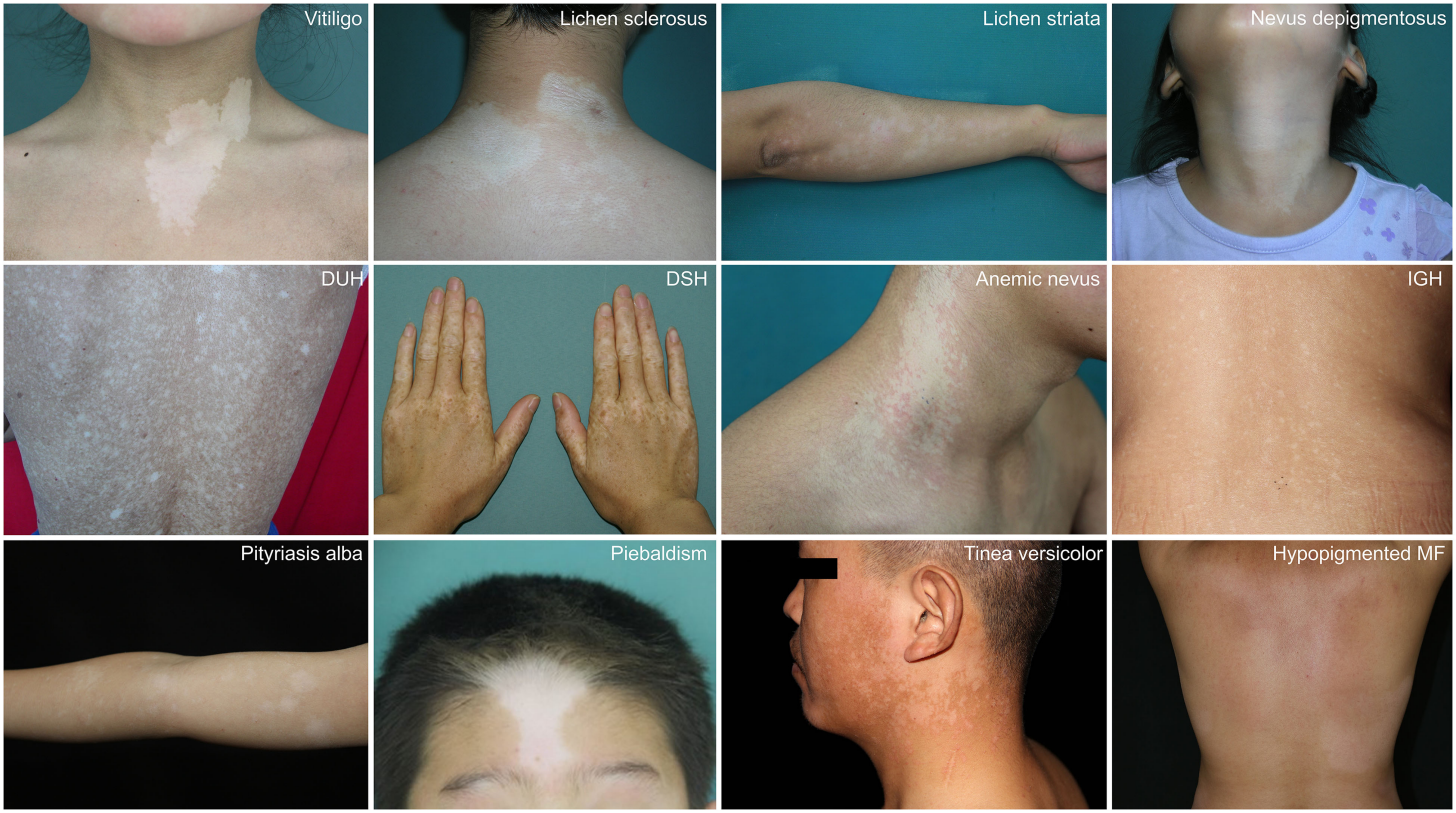
Representative clinical images of vitiligo and other hypopigmentary disorders. DUH, dyschromatosis universalis hereditaria; DSH, dyschromatosis symmetrica hereditaria; IGH, idiopathic guttate hypomelanosis; Hypopigmented MF, mycosis fungoides.

This study included a total of 13,322 images from 2,974 patients. The vitiligo group comprised 3108 images from 1,133 patients, which were split into a training set of 2,176 images and a validation set of 932 images. The non-vitiligo group consisted of 10,214 images from 1,841 patients, with 7,150 used for training and 3,064 for validation (**Figure 3**, **Table 2**). The dataset covers ten types of hypopigmentary disorders that require differential diagnosis from vitiligo. Representative examples of vitiligo and the other differential diseases are presented in **Figure 4**.

**Table 2:** Distribution of training and validation datasets for vitiligo and its differential diagnoses.

Types of skin disease	Patients, n	Training images, n	Validation images, n	Total images, n
Vitiligo	1,133	2,176	932	3,108
Lichen sclerosus	554	1,293	554	1,847
Lichen striatus	516	1,538	659	2,197
Nevus depigmentosus	78	686	294	980
Hereditary dyschromatosis	38	630	270	900
Nevus anemicus	180	642	275	917
Idiopathic guttate hypomelanosis	100	565	242	807
Pityriasis alba	100	385	165	550
Piebaldism	30	359	154	513
Tinea versicolor	200	744	319	1,063
Hypopigmented MF	45	308	132	440
Total	2,974	9,326	3,996	13,322

### 3.2 Accuracy of characteristic classification

As shown in **Supplementary Figure 2**, the model demonstrated varying accuracy in the multi-label classification of eight vitiligo lesion characteristics, with overall accuracy ranging from 52.93% to 88.12%. It achieved the highest performance in recognizing typical location (88.12%) and edge morphology (86.78%). In contrast, the number of lesions (52.93%) was the least accurate among all characteristics evaluated.

### 3.3 Performance of the diagnostic model

The proposed diagnostic model was evaluated for its ability to differentiate vitiligo from other hypopigmentary disorders, both as a binary classifier (vitiligo vs. non-vitiligo) and as a multi class discriminator across all 11 conditions.

#### 3.3.1 Confusion matrix and classification performance

**Figure 5:**
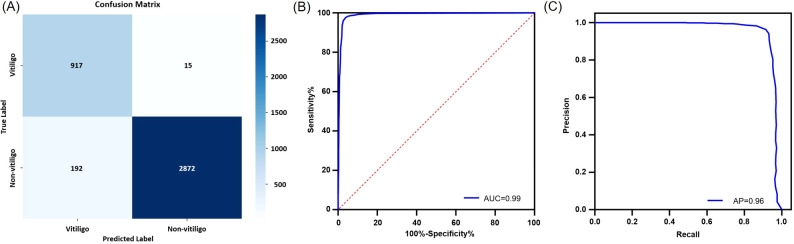
Performance of the model for vitiligo diagnosis. **(A)** Confusion matrix showing the classification results for vitiligo and other hypopigmentary disorders. **(B)** Receiver Operating Characteristic (ROC) curve with an Area Under the Curve (AUC) of 0.99. **(C)** Precision-Recall (PR) curve illustrating the trade-off between precision and recall, with an average precision (AP) score of 0.96.

As shown in **Figure 5A**, the model attained an accuracy of 94.82%, with a sensitivity of 98.29% and a specificity of 93.73% for vitiligo detection. These results indicate that the model effectively identifies true vitiligo cases while reliably excluding non-vitiligo conditions, which is critical for reducing misdiagnosis in clinical practice, as false positives may lead to unnecessary treatments and patient anxiety, while false negatives can delay appropriate therapy and allow disease progression.

#### 3.3.2 ROC curve analysis

The ROC curve for the binary classification of vitiligo versus all other disorders is shown in **Figure 5B**, yielding an excellent AUC of 0.9906 (95% CI: 0.9844–0.9968). To fully evaluate the model’s discriminative capability across the entire spectrum of diseases, a multi-class ROC analysis was performed. The model demonstrated strong diagnostic performance for all 11 hypopigmentary disorders, with the highest AUC observed for vitiligo. The AUCs for other diseases ranged from 0.68 to 0.94, as detailed in the **Supplementary Figure 3**. These results affirm the model’s high overall accuracy and its potential to support differential diagnosis in clinical settings where visual assessment alone may be challenging.

#### 3.3.3 Precision-Recall curve and stability under imbalance

The precision-recall curve (**Figure 5C**) yielded an average precision (AP) score of 0.96, highlighting the model’s robust performance even when dealing with a limited number of positive samples (vitiligo cases). This indicates that the model maintains high precision across varying recall levels, thereby reducing the likelihood of overdiagnosis or unnecessary referrals—a critical advantage in contexts with low disease prevalence or imbalanced datasets.

### 3.4 Examples of clinical applications

**Figure 6:**
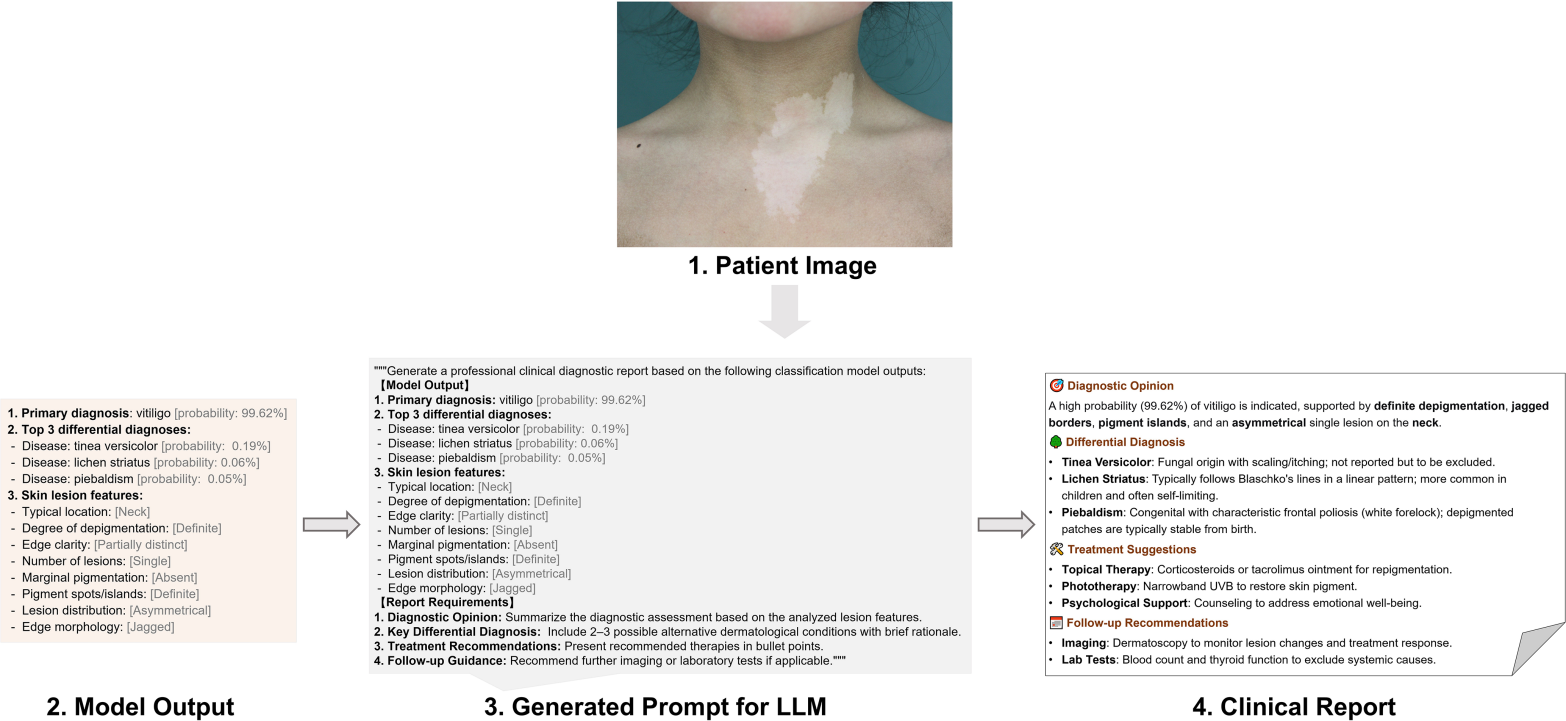
A proposed workflow for clinical application of the AI model. The process begins with (1) a clinical image of a patient’s lesion. The image is input into our model, which generates (2) a diagnostic output, including the probability of vitiligo or other conditions. This output is then automatically converted into (3) a structured prompt, which is used to generate (4) a comprehensive clinical report.

A representative case was used to illustrate the model’s clinical utility. Based on the model outputs, the generated clinical report indicated a high probability of vitiligo (99.62%), listed differential diagnoses like tinea versicolor, lichen striatus, and piebaldism, outlined treatment plans including topical medication, phototherapy, and psychological counseling, and provided follow-up recommendations such as dermatoscopic imaging and laboratory tests. This case demonstrates the model’s ability to deliver structured and clinically valuable diagnostic information, supporting accurate diagnosis, differentiation from other hypopigmentary disorders, and informed treatment planning (**Figure 6**).

### 3.5 Quality assessment of LLM-generated clinical reports

To systematically evaluate the clinical quality of the LLM-generated reports, a blinded assessment was conducted by three vitiligo specialists on a randomly selected 30 cases (15 vitiligo, 15 non−vitiligo). Each report was rated on a 5−point Likert scale across four dimensions corresponding to the report structure. As summarized in **Table 3**, the mean scores ranged from 4.13 ± 0.28 to 4.52 ± 0.35 across the four dimensions, with the highest score for diagnostic opinion accuracy. The intraclass correlation coefficients (ICC) ranged from 0.71 to 0.80, indicating moderate to good inter−rater agreement. These findings demonstrate that the LLM-generated reports are clinically reliable and consistent across multiple evaluators.

**Table 3:** Quality assessment of LLM-generated clinical reports by three vitiligo specialists.

Assessment dimension	Mean ± SD	ICC	95% CI
Diagnostic opinion accuracy	4.52 ± 0.35	0.80	0.65 – 0.89
Differential diagnosis plausibility	4.13 ± 0.28	0.74	0.57 – 0.85
Treatment appropriateness	4.46 ± 0.33	0.71	0.52 – 0.83
Follow-up plan reasonableness	4.51 ± 0.29	0.77	0.60 – 0.87

Mean scores were calculated from 90 ratings per dimension (30 cases × 3 dermatologists). ICC: two−way random, absolute−agreement intraclass correlation coefficient for single raters.

### 3.6 Outcomes for the model compared to dermatologists

**Figure 7:**
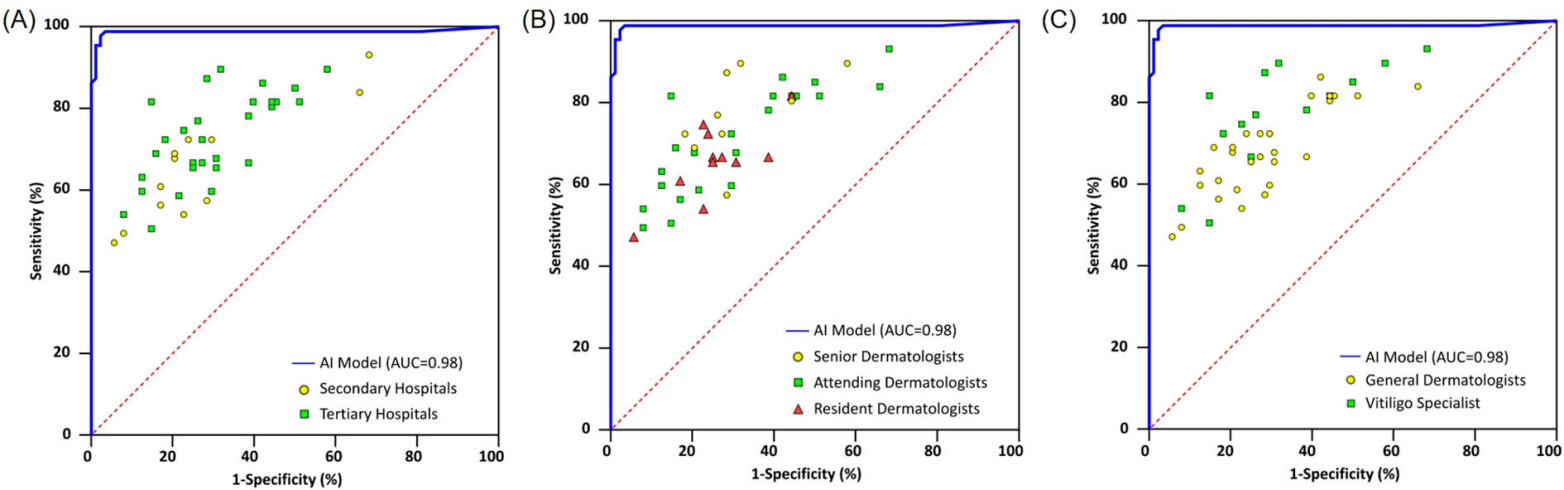
Comparison of diagnostic performance between the AI model and dermatologist subgroups. The sensitivity and specificity of the proposed AI model and various groups of dermatologists were evaluated on an independent test set of 175 clinical images (87 vitiligo, 88 non-vitiligo). The AI model achieved an AUC of 0.98. **(A)** Comparison between the AI model and dermatologists stratified by hospital tier (secondary vs. tertiary hospitals). **(B)** Comparison between the AI model and dermatologists stratified by professional seniority (resident, attending, and senior dermatologists). **(C)** Comparison between the AI model and dermatologists stratified by specialty background (general dermatologists vs. vitiligo specialists).

In an independent evaluation based on 175 clinical images, the AI model developed in this study demonstrated excellent diagnostic performance (AUC = 0.98, sensitivity=98.85%, specificity=96.59%), significantly surpassing the overall performance of the dermatologists (**Figure 7**). Further subgroup analyses revealed performance variations among the dermatologists. When stratified by hospital tier, the sensitivity of dermatologists from tertiary hospitals (72.97%) was higher than that of those from secondary hospitals (65.33%) (**Figure 7A**, **Table 4**). In the analysis based on professional seniority, diagnostic sensitivity increased with higher seniority (resident: 65.62%; attending: 70.81%; senior: 77.27%), though these differences didn’t reach statistical significance (**Figure 7B**, **Table 5**). Notably, when comparing dermatologists by specialty, vitiligo specialists exhibited significantly higher diagnostic sensitivity than general dermatologists (77.26% vs. 67.74%, *P* = 0.013). Nevertheless, no statistically significant differences were observed between the two groups in terms of specificity or overall accuracy (**Figure 7C**, **Table 6**).

**Table 4:** Comparison of diagnostic performance between dermatologists stratified by hospital tier.

Variable	Secondary Hospitals (n=12)	Tertiary Hospitals (n=31)	*P* value
Sensitivity, % (95% CI)	65.33 (56.52,74.13)	72.97 (69.02,76.92)	0.061
Specificity, % (95% CI)	72.73 (60.10,85.36)	69.54 (64.80,74.27)	0.538
Accuracy, % (95% CI)	69.05 (65.85,72.25)	71.24 (69.52,72.96)	0.184

CI, confidence interval. P values were derived from independent two-sample t-tests. P-value < 0.05 was considered statistically significant.

**Table 5:** Comparison of diagnostic performance between dermatologists stratified by professional seniority.

Variable	Senior Dermatologists (n=9)	Attending Dermatologists (n=23)	Resident Dermatologists (n=11)	*P* value
Sensitivity, % (95% CI)	77.27 (68.99,85.54)	70.81 (65.28,76.35)	65.62 (59.30,71.95)	0.097
Specificity, % (95% CI)	68.56 (59.03,78.09)	69.32 (61.64,77.00)	74.28 (67.43,81.12)	0.620
Accuracy, % (95% CI)	72.89 (68.60,77.18)	70.06 (67.90,72.22)	69.97 (67.62,72.33)	0.295

CI, confidence interval. P values were derived from a one-way ANOVA. P-value < 0.05 was considered statistically significant.

**Table 6:** Comparison of diagnostic performance between dermatologists stratified by specialty background.

Variable	General Dermatologists (n=29)	Vitiligo Specialist (n=14)	*P* value
Sensitivity, % (95% CI)	67.74 (63.75,71.73)	77.26 (69.83,84.69)	0.013
Specificity, % (95% CI)	67.94 (66.42,76.84)	71.63 (57.75,78.13)	0.456
Accuracy, % (95% CI)	69.69 (68.21,71.18)	72.57 (69.09,76.05)	0.121

CI, confidence interval. P values were derived from independent two-sample t-tests. P-value < 0.05 was considered statistically significant.

## 4 Discussion

This study successfully developed an intelligent diagnostic system integrating deep learning with an LLM for discriminating between vitiligo and ten other hypopigmentary skin disorders. Our results demonstrate that the system not only achieves high diagnostic accuracy (AUC 0.99) but, more importantly, addresses the “black-box” challenge inherent in conventional AI models by simultaneously providing visual explanations based on clinical characteristics and generating structured clinical reports. This dual capability offers clinicians comprehensive, transparent, and clinically actionable diagnostic and decision-making support.

Our model demonstrated excellent diagnostic performance, with an AUC of 0.9906 (95% CI: 0.9844–0.9968) on a large-scale dataset of 2,974 patients, surpassing that of previous smaller-scale studies, which reported AUCs of 0.91 and 0.89 using ResNet152 and DenseNet121 on dermoscopic images for binary vitiligo detection (13). Notably, the model also achieved robust discriminative capability in the multi-class setting across all 11 hypopigmentary disorders. The observed spectrum of AUCs (0.68 to 0.94) realistically reflects the varying clinical presentation and inherent diagnostic difficulty among different conditions. Importantly, the model attained its highest performance for vitiligo, fulfilling the primary diagnostic objective. For vitiligo, the high sensitivity of 98.29% ensures that the majority of vitiligo patients are correctly identified, preventing delays in treatment, as early intervention is known to improve stabilization and repigmentation outcomes (16). Meanwhile, the high specificity of 93.73% minimizes unnecessary treatments and associated costs for patients with non-vitiligo conditions (17). For other disorders, the model provides valuable probabilistic differentiation, effectively narrowing down the diagnostic candidates, a key advantage in clinical scenarios where visual assessment is challenging. Furthermore, the AP value of 0.96confirms the model’s robustness against class imbalance. The variation in per-disease AUC is also attributable to the natural imbalance in real-world data availability, with rarer conditions presenting a greater learning challenge. Compared to existing research, our model not only maintains high precision across a broader spectrum of differential diagnoses but also addresses the common limitations of limited sample size and poor generalizability.

A major obstacle to the clinical adoption of AI in dermatology is the “black-box” nature of many high-performance models (18, 19). These systems typically output only a disease label and probability score without explaining the reasoning behind their predictions or offering further diagnostic guidance, thereby limiting their utility in complex clinical decision-making (20, 21). Our study directly addresses this challenge through two key innovations.

First, we introduced a visual explanation mechanism based on clinical characteristics. Through its multi-task architecture, the model extracts eight key clinical characteristics for differential diagnosis. To present these findings intuitively, we present seven quantifiable characteristics (including typical location, degree of depigmentation, and edge clarity) in a radar chart. This design makes the model’s decision-making process transparent. For example, when a lesion is diagnosed as vitiligo, clinicians can verify whether the model has identified supporting evidence such as “definite depigmentation”, “indistinct borders”, or “islands of pigmentation”. This alignment with clinical diagnostic logic significantly enhances the model’s credibility and acceptability. Notably, typical location and border morphology are among the most discriminative visual clues. The model’s high accuracy in identifying them (88.12% and 86.78%, respectively) validates its ability to capture critical diagnostic information. The low accuracy for lesion count (52.93%) likely reflects both its limited clinical discriminative value and the inherent difficulty of quantifying lesions from partial-body photographs. Future iterations may simplify or remove this feature.

Second, this study is the first to integrate an LLM (DeepSeek-R1) into the AI diagnostic workflow for skin diseases to generate structured, clinical reports. While traditional AI models terminate with a disease label, our system uses the image analysis results (including characteristic classifications and diagnostic probabilities) as input for a carefully designed prompt template. This guides the LLM to generate a comprehensive report encompassing diagnostic opinions, differential diagnoses, treatment suggestions, and follow-up plans. Blinded evaluation of the LLM-generated reports by three vitiligo specialists on 30 independent cases confirmed their clinical reliability, with mean scores ranging from 4.13 to 4.52 (out of 5) and ICC values ranging from 0.71 to 0.80. This marks a fundamental evolution of the AI’s role—from a passive “classifier” to an active “clinical decision partner”. The provision of differential reasoning and treatment recommendations directly addresses the core needs of clinicians, enabling AI to be truly integrated into the diagnostic and therapeutic loop, and offering greater clinical utility than a single classification model.

The AI model developed in this study demonstrated superior performance in vitiligo diagnosis compared to the clinician cohort, which aligns with the observed trend of AI applications in dermatological image analysis (10, 22). Subgroup analysis among dermatologists indicated numerically higher sensitivity for those from tertiary hospitals or with greater seniority, though the differences were not statistically significant, which may be attributed to sample size limitations. Notably, vitiligo specialists exhibited significantly higher sensitivity than general dermatologists, underscoring the clear value of specialized training and disease-specific clinical experience in enhancing diagnostic accuracy (3, 23). This performance gap suggests that the AI model could serve as an effective tool to assist non-specialists, thereby helping to narrow diagnostic disparities across different clinical settings (24, 25).

The intelligent diagnostic system developed in this study holds significant clinical potential. In primary care, it assists general practitioners in making accurate differential diagnoses, reducing misdiagnosis and enabling timely referral of suspected vitiligo. In specialized dermatology clinics, it serves as an efficient screening and triage tool, helping experts focus on complex cases by managing common ones and promoting diagnostic consistency through standardized outputs. Its intuitive radar charts and clear reports can be integrated into electronic health records, aiding patient understanding and potentially improving treatment adherence. Importantly, by providing reliable diagnostic support where histopathological confirmation is delayed or unavailable, the system helps bridge the gap between specialized expertise and frontline clinical needs. This is especially valuable in developing countries and remote regions, where it can deliver low-cost, high-quality preliminary skin diagnostics via mobile platforms.

Several limitations of this study should be noted. First, although ten other hypopigmentary conditions were included, some rare conditions had limited sample sizes, such as Hypopigmented MF, which may affect the generalizability of the model. Second, the current system relies solely on clinical images and does not incorporate multimodal data such as patients’ history or laboratory results, which may be crucial for complex cases. Third, although the LLM-generated reports are logically coherent, their clinical reliability requires further validation in prospective studies. Fourth, owing to the retrospective multi−center design, some image heterogeneity was not fully controlled; standardized acquisition protocols should be implemented in future work. Fifth, our cohort was derived from a Chinese population (predominantly Fitzpatrick II–III), and performance in darker skin types (IV–VI) requires further validation. Other limitations include selection bias inherent to the retrospective design, the absence of real-workflow metrics (report generation time, clinician acceptance), and the lack of systematic robustness testing on poor-quality or atypical images.

## Conclusion

In conclusion, we present a high-performance, interpretable AI-assisted diagnostic system for differentiating vitiligo from multiple hypopigmentary disorders. By combining deep learning–based feature extraction with LLM-driven reporting, it shifts the paradigm from “black-box” classification to “white-box” decision support. This tool not only addresses the clinical challenge of vitiligo diagnosis but also paves a new, clinically meaningful pathway for medical AI. Future efforts will focus on expanding sample diversity, integrating multimodal data, and conducting prospective trials to further validate the system’s efficacy in real-world settings. With ongoing refinement, such systems are poised to become invaluable aids for dermatologists, advancing the field toward more precise and intelligent healthcare.

## Data Availability

The raw data supporting the conclusions of this article will be made available by the authors, without undue reservation.
