# Supplementary figures and images for: A two-stage workflow for vitiligo diagnosis: clinical characteristic classification and large language model (LLM)–based report generation
Source: Front Immunol. 2026 Jun 1;17:1853327. doi: 10.3389/fimmu.2026.1853327 (PMC13265523; doi:10.3389/fimmu.2026.1853327)

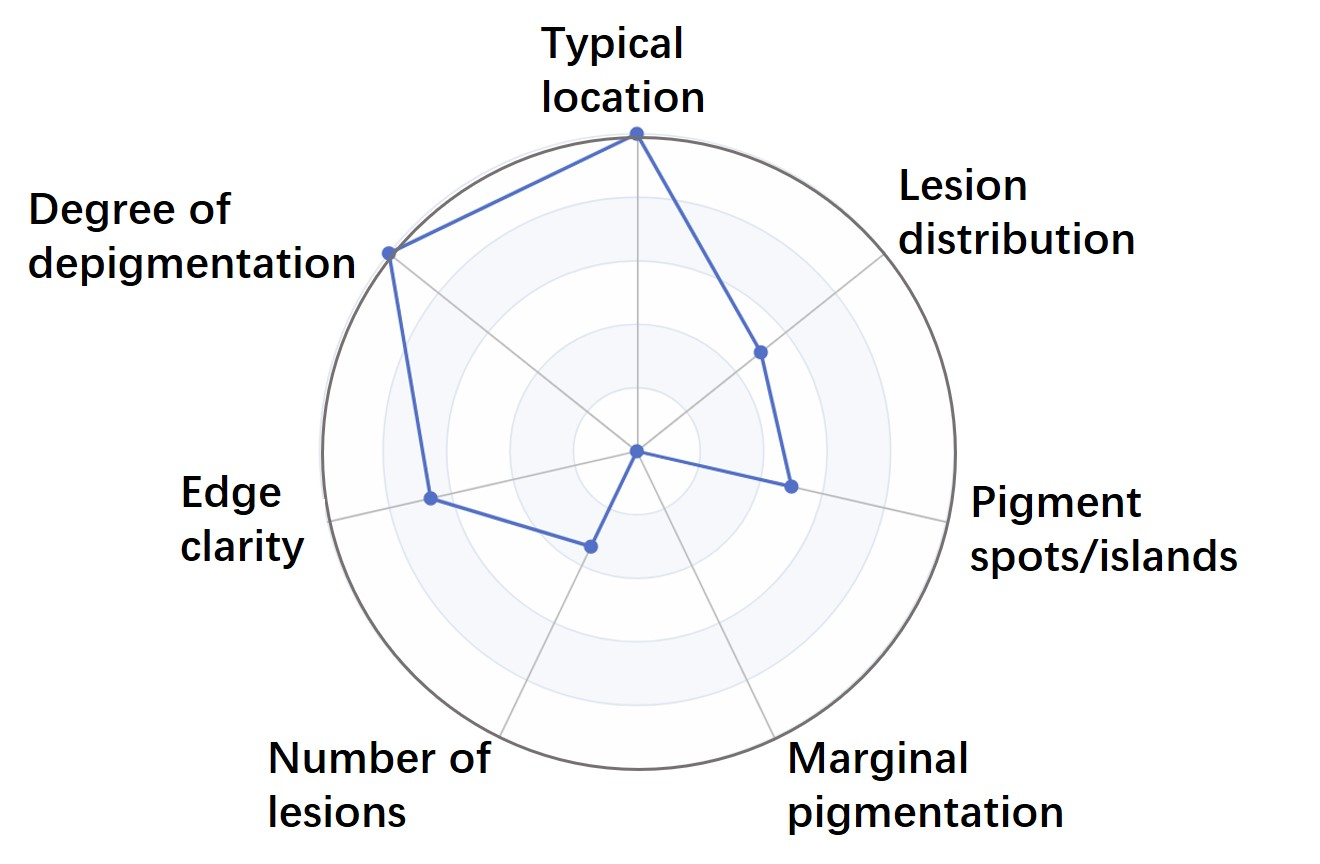

Supplement: Supplementary file 1 [file Image1.tiff]

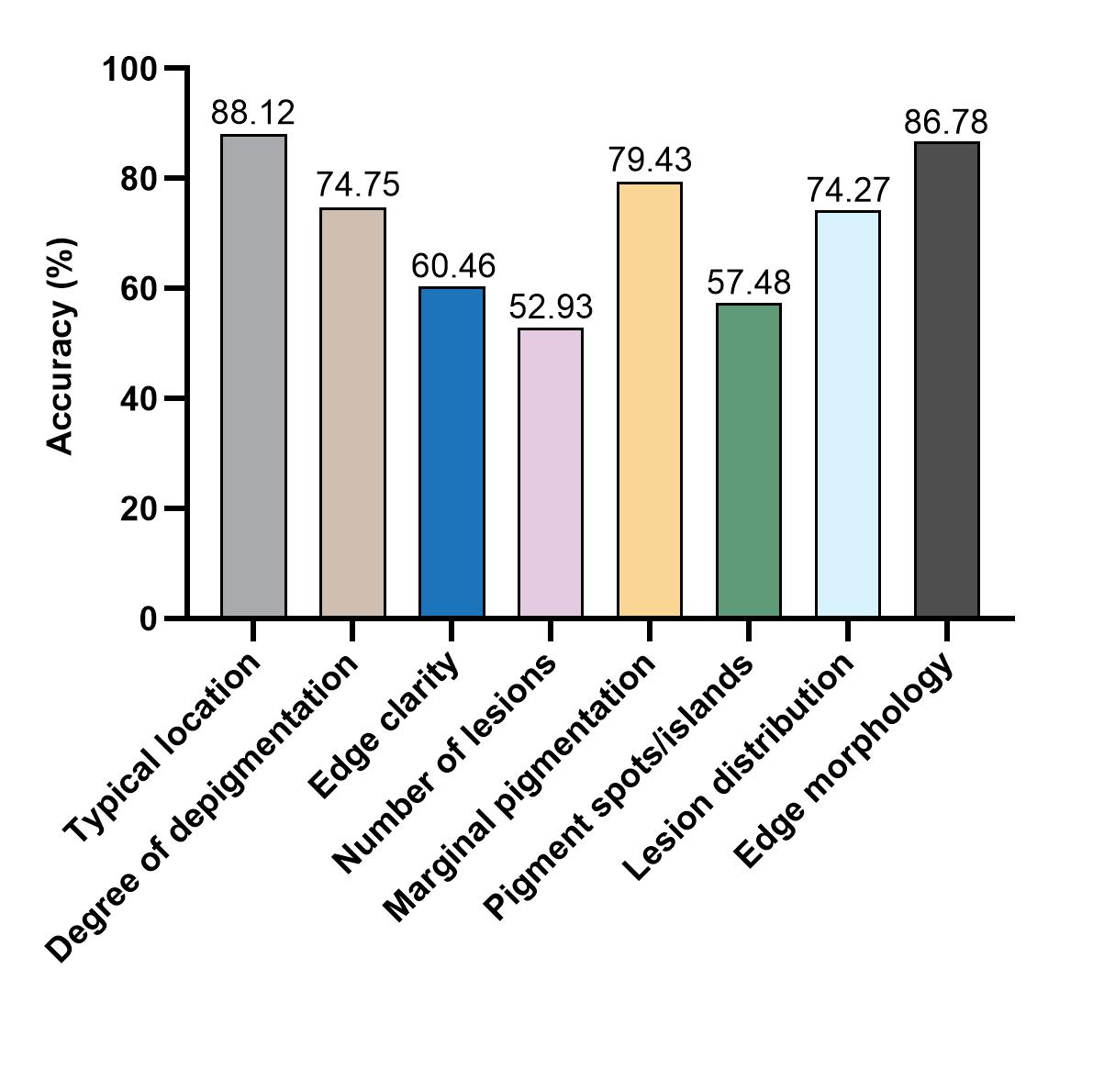

Supplement: Supplementary file 2 [file Image2.tiff]

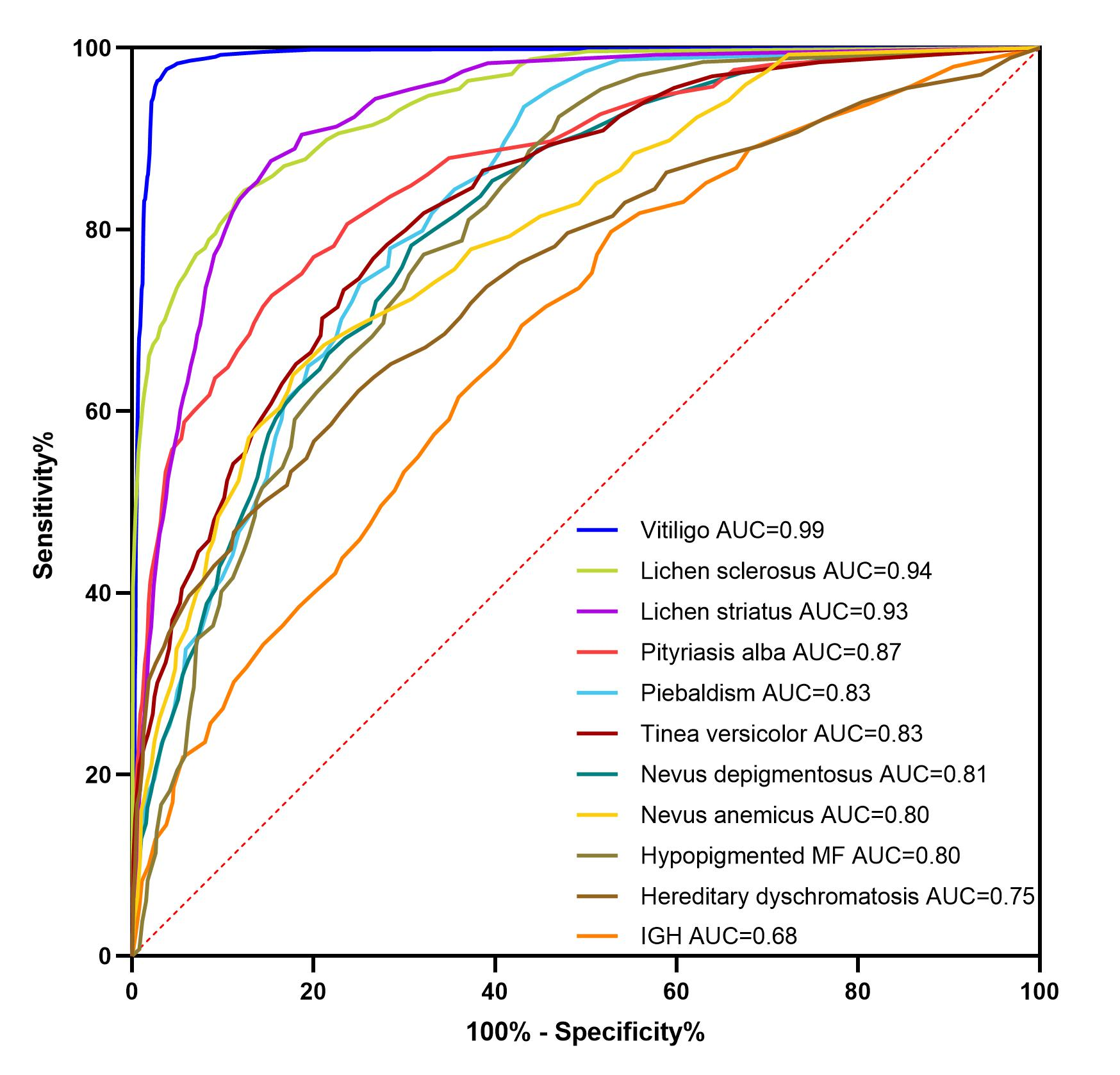

Supplement: Supplementary file 3 [file Image3.tiff]
