# Supplementary material for: A two-stage workflow for vitiligo diagnosis: clinical characteristic classification and large language model (LLM)–based report generation
Source: Front Immunol. 2026 Jun 1;17:1853327. doi: 10.3389/fimmu.2026.1853327 (PMC13265523; doi:10.3389/fimmu.2026.1853327)
Supplement: Supplementary file 4 [file Table1.docx]

**Supplementary information**

**Supplementary Methods**

**Details of annotated characteristics and their clinical significance**

1. **Typical location**: The anatomical distribution of lesions is a primary clue for differentiation. Vitiligo preferentially affects sun-exposed areas (face, neck, hands), friction-prone sites (waist, axillae, groin), and mucosal membranes. This differs markedly from lichen sclerosus (predominantly affects the anogenital region); nevus depigmentosus (typically segmental and stable from birth); and nevus anemicus (often on trunk). Pityriasis alba most commonly involves the face; piebaldism characteristically affects the forehead (central, triangular patch) and ventral trunk; tinea versicolor favors the trunk (chest, back) and proximal extremities; and hypopigmented MF often occurs on sun-protected areas such as the trunk, buttocks, and thighs. While not definitive alone, this characteristic significantly narrows the differential diagnosis.
2. **Degree of depigmentation:** Vitiligo typically shows complete pigment loss, yielding a distinct milky-white color. In contrast, conditions like nevus depigmentosus and idiopathic guttate hypomelanosis usually present with incomplete or partial loss. Similarly, pityriasis alba and tinea versicolor exhibit incomplete hypopigmentation, and hypopigmented MF lesions are often subtly pale. Piebaldism, however, can present with areas of complete depigmentation. This clear difference provides a quantifiable, high-value characteristic for AI-based discrimination.
3. **Edge clarity**: The clarity of a lesion’s border reflects disease activity in vitiligo. Stable lesions have sharp edges, while active ones show blurred margins, sometimes with surrounding inflammation. By comparison, static conditions like nevus depigmentosus and nevus anemicus maintain irregular but consistent borders. Pityriasis alba is characterized by ill-defined, ‘fuzzy’ borders; tinea versicolor lesions are usually well-demarcated; and hypopigmented mycosis fungoides patches may have variable but generally discrete margins. Pixel gradient analysis at the lesion edge can help objectively quantify this characteristic.
4. **Number of lesions**: The lesion count provides supportive diagnostic evidence. Vitiligo often presents with multiple (and sometimes numerous) lesions. In contrast, nevus depigmentosus and nevus anemicus are typically solitary. Pityriasis alba, tinea versicolor, and hypopigmented mycosis fungoides commonly present with multiple lesions. Piebaldism usually features a limited number of stable, congenital patches. While less weighted than other characteristics, it contributes valuable context to the overall diagnostic assessment.
5. **Marginal pigmentation**: A hyperpigmented rim at the lesion edge strongly suggests vitiligo, reflecting melanocyte repigmentation during active disease. This sign is rare in other hypopigmentary disorders and, if detected, greatly raises confidence in a vitiligo diagnosis.
6. **Pigment spots/islands**: Pigmentation spots within a depigmented patch are highly specific to vitiligo and indicate regenerative activity. This characteristic is not seen in the other differential diagnoses listed.
7. **Lesion distribution**: The overall pattern of lesion distribution is critical for differentiation. Vitiligo can exhibit symmetric, generalized, or uniquely segmental patterns following a dermatome. This segmental pattern helps distinguish it from linear lichen planus, which follows Blaschko’s lines. Pityriasis alba is often clustered on the face; piebaldism follows a characteristic pattern involving the central forehead, ventral trunk, and extremities; tinea versicolor is typically scattered or confluent on the seborrheic areas; and hypopigmented MF lesions can be widespread in a ‘non-segmental’ pattern. Recognizing “segmental” versus “symmetric” or “scattered” patterns is therefore key to differentiating vitiligo from its mimics.
8. **Edge morphology**: Border shape offers further diagnostic context. Vitiligo edges may be smooth, irregular, geographic, or jagged—the last often signaling active spread. Nevus depigmentosus can also appear jagged or “splash-like” but remains static; idiopathic guttate hypomelanosis shows smooth, round borders. Pityriasis alba edges are typically indistinct and feathered; tinea versicolor lesions are often oval or round with fine scaling at the border; hypopigmented MF may show slightly atrophic or wrinkled surfaces with subtle, irregular borders. While less specific than other characteristics, edge morphology adds useful nuance to activity assessment and differential diagnosis.

**Model training implementation details**

The experiments were performed on a workstation equipped with an NVIDIA Quadro RTX 3000 graphics card (6GB memory) and implemented using the PyTorch 2.0 framework. Before training, all images were standardized and preprocessed, keeping the aspect ratio to adjust the short edge to 256 pixels, the center was cropped to 224×224 area, and the pixel value was normalized to the range of [0,1].

In the initial stage, the parameters of the first 8 layers of ViT backbone were frozen, and only the last 4 layers of Transformer and classification head were optimized. The learning rate was set to 1e-4. In the second stage, all parameters were thawed for end-to-end fine-tuning, and the learning rate was reduced to 1e-5. Adam algorithm (β1=0.9, β2=0.999) was used for optimization, with a batch size of 64 and a maximum training round of 300.

1. Loss Function: Loss function design

This project designs the multi-task learning loss function, which includes the following three parts.

1.1 Multi - class classification and multi-label classification loss

For each characteristic (8 in total), the cross-entropy loss function was used to calculate the classifier loss. Suppose that the predicted probability distribution of the first characteristic is, and the true label is, then the cross-entropy loss of the first characteristic is:$\text{i}\hat{y}_{i}y_{i}\text{i}L_{i}$

$$L_{i}=-\sum_{c} y_{i,c}\log(\hat{y}_{i,c})$$

Where, c represents the category index, is the indicator value (0 or 1) of class c in the true label of the ith characteristic, and is the probability that the ith characteristic belongs to class c predicted by the model.$y_{i,c}\hat{y}_{i,c}$

1.2 Final Diagnostic loss

For the diagnosis of vitiligo, we used the binary cross-entropy loss function to calculate the loss. $\hat{p}$ Assuming that the predicted probability of a vitiligo diagnosis is, and the true label is (0 or 1), the binary cross-entropy loss of a vitiligo diagnosis is:$pL_{d}$

$$L_{d}=-p\log\left( \hat{p} \right)-\left( 1-p \right)\log\left( 1-\hat{p} \right)$$

1.3 Total loss (total loss)

The total loss is obtained by the weighted sum of each loss. $\alpha_{i}$Assuming that the weight of each characteristic loss is, and the weight of vitiligo diagnosis loss is, the total loss is:$\beta L_{total}$

$$L_{total}=\sum_{i=1}^{8} \alpha_{i}L_{i}+\beta L_{d}$$

Where, and are the preset weight parameters used to balance the importance of different loss terms.$\alpha_{i}\beta$

2. Training process

In order to avoid over-fitting to accelerate training, we adopt a phased training strategy. In the initial phase, we freeze all parameters except the last four encoder blocks in the ViT backbone network and only train the clinical concept prediction head and the last four encoder blocks. In the subsequent stage, all parameters of the ViT backbone network were selected to be thawed and the learning rate was reduced for fine tuning.

**Detailed implementation and specifications of the three-level cascade model for differential diagnosis of hypopigmentary disorders**
**(1) ViT-B/16 characteristic extraction backbon**e

The input RGB image of size 224×224 pixels is divided into 196 non-overlapping 16×16 pixel patches. Each patch is flattened and linearly projected into a 768-dimensional embedding vector, with learnable position embeddings added to retain spatial information. The sequence of patch embeddings, prepended with a special [CLS] token, is then fed into a 12-layer Transformer encoder. Each encoder layer consists of Layer Normalization applied before each sub-block, Multi-Head Self-Attention with 12 attention heads (each of dimension 64), a Feed-Forward Network with a hidden layer dimension of 3072, and residual connections around both the Multi-Head Self-Attention and Feed-Forward Network sub-blocks to ensure stable training. The final hidden state corresponding to the [CLS] token, with a dimension of 768, is extracted as the global image characteristic representation for subsequent tasks.

**(2) Multi-task characteristic reasoning module**

This module processes the global characteristics from the ViT backbone and performs parallel predictions for individual characteristics. It includes seven independent binary or multiclass classification heads (characteristic_head1 to characteristic_head7), each composed of a linear layer that maps the 768-dimensional input to the required number of classes, followed by a Softmax activation function to predict the likelihood of each of the seven key clinical characteristics. Additionally, a dedicated multi-label classification head (characteristic_head8) maps the 768-dimensional input to an 8-dimensional vector representing the probabilities of different edge morphologies (e.g., smooth, irregular, jagged) via a linear layer and Sigmoid activation.

**(3) Diagnostic Probability Prediction**

The outputs from the eight characteristic heads—comprising seven classification results and one multi-label result—are concatenated into a single 30-dimensional characteristic vector. This vector is then passed through a two-layer fully connected network, where the input layer consists of 30 dimensions, the hidden layer contains 64 neurons with ReLU activation, and the output layer comprises a single neuron with Sigmoid activation. The final output is the diagnostic probabilities distribution of 11 hypopigmentary disorders.

**A professional clinical diagnostic report template based on the following classification model outputs.**

【Model Output】

1. Primary diagnosis: Disease [prediction results]

2. Top 3 differential diagnoses:

- Disease: [prediction results]

- Disease: [prediction results]

- Disease: [prediction results]

3. Skin lesion characteristics:

   - Typical location: [prediction results]

   - Degree of depigmentation: [prediction results]

   - Edge clarity: [prediction results]

- Number of lesions: [prediction results]

   - Marginal pigmentation: [prediction results]

   - Pigment spots/islands: [prediction results]

   - Lesion distribution: [prediction results]

   - Edge morphology: [prediction results]
【Report Requirements】

1. Diagnostic conclusion: Summarize the diagnostic assessment based on the analyzed lesion characteristics.

2. Differential diagnosis: Include 2–3 possible alternative dermatological conditions with brief rationale.

3. Treatment Recommendations: Present recommended therapies in bullet points.

4. Follow-up Guidance: Recommend further imaging or laboratory tests if applicable."""

**Supplementary Figure legends:**

**Figure S1. Radar chart of characteristic visualization**

**Figure S2. Classification accuracy of 8 clinical characteristics**

**Figure S3. Multi-class receiver operating characteristic (ROC) curves of the diagnostic model.** The model was evaluated on the independent validation set for its ability to discriminate among all 11 hypopigmentary disorders. The area under the curve (AUC) for each disease is indicated in the legend, ranging from 0.68 to 0.99, with vitiligo achieving the highest discriminative performance.

**Table S1. Clinical characteristic quantification criteria**

| **Clinical characteristics** | **Quantification level**  **(0→N)** | **Severity mapping relationship** |
| --- | --- | --- |
| Typical location | 0-1 | No (0) → Yes (1) |
| Degree of depigmentation | 0-2 | Absent (0) →Indeterminate (1) → Definite (2) |
| Edge clarity | 0-3 | Cannot be assessed (0) →Partially distinct (1) →Indistinct (2) → Distinct (3) |
| Number of lesions | 0-3 | Cannot be assessed (0) → Single (1) → 2-3 (2) → ≥4 (3) |
| Marginal pigmentation | 0-2 | Absent (0) →Indeterminate (1) → Definite (2) |
| Pigment spots/islands | 0-2 | Absent (0) →Indeterminate (1) → Definite (2) |
| Lesion distribution | 0-2 | Cannot be assessed (0) → Asymmetric (1) → Symmetrical (2) |
